# *Hsp101-1* Orchestrates Thermotolerance in Rice via Pre-Activated Transcriptional Networks and Modular Cross-Tissue Coordination

**DOI:** 10.3390/genes16091039

**Published:** 2025-08-31

**Authors:** Hang Yu, Liqun Jiang, Bingrui Sun, Qing Liu, Xingxue Mao, Jing Zhang, Pingli Chen, Wenfeng Chen, Chen Li, Shuwei Lyu

**Affiliations:** 1 Rice Research Institute, Guangdong Academy of Agricultural Sciences, Guangzhou 510640, China; yuhang@gdaas.cn (H.Y.); jiangliqun@gdaas.cn (L.J.); sunbingrui2003@163.com (B.S.); liuqing198504@126.com (Q.L.); maox@gdaas.cn (X.M.); zhangj6800@126.com (J.Z.); pingzi0826@163.com (P.C.); 18-cwf@163.com (W.C.); lichen@gdaas.cn (C.L.); 2 Key Laboratory of Genetics and Breeding of High Quality Rice in Southern China (Co-Construction by Ministry and Province), Ministry of Agriculture and Rural Affairs, Guangzhou 510640, China; 3 Guangdong Key Laboratory of Rice Science and Technology, Guangzhou 510640, China; 4 Guangdong Rice Engineering Laboratory, Guangzhou 510640, China

**Keywords:** *Hsp101-1*, thermotolerance, transcriptome stability, WGCNA, modular networks, rice

## Abstract

**Background/Objectives:** Rice production faces threats from rising temperatures, demanding thermotolerant varieties. This study characterizes transcriptomic dynamics and identifies *Hsp101-1* (*heat shock protein 101-1*)-associated gene regulatory modules in rice under reproductive-stage heat stress. **Methods:** Transcriptomics and WGCNA (weighted gene co-expression network analysis) were conducted in flag leaves and spikelets for wild-type (WT) and *Hsp101-1*-overexpressing (OE) lines under 40 °C stress at six time points (0–24 h) to reveal the change in gene expressions. **Results:** The number of DEGs (differentially expressed genes) revealed substantial pre-existing differences in WT and OE lines. Pre treatment, OE flag leaves showed 545 upregulated and 676 downregulated DEGs versus WT leaves. Post heat shock, the number of DEGs in flag leaves and spikelets was significantly reduced by 70–80%. KEGG enrichment of common DEGs across time points showed both WT and OE flag leaves enriched for ribosome biogenesis, ribosomes, and chaperones/folding catalysts. WGCNA identified that the MEdarkslateblue module correlated negatively with WT and positively with OE flag leaves. The MEturquoise module was suppressed at 1 h but activated by 8 h. Spikelet analysis identified the MElightpink4 module (negative correlation with WT, positive with OE) and a similarly dynamic MEturquoise module. Venn analysis identified 76 shared module genes, 71 of which were upregulated in the OE line, indicating that Hsp101-1 activates common protective targets. Hsp101-1’s expression in the WT line was low basally, significantly upregulated at 1–8 h post shock, and returned to low levels by 24 h. **Conclusions:** *Hsp101-1* enhances thermotolerance by (1) constitutively pre-stabilizing transcriptomic networks and reducing transcriptional fluctuations under heat stress and (2) modularly coordinating tissue-specific responses, providing a climate resilience framework.

## 1. Introduction

Rice (*Oryza sativa* L.) serves as a main calorie source for over half of the global population [1]. However, rising global temperatures driven by climate change pose a significant threat to rice productivity [2,3,4]. Such thermal stress disrupts critical physiological and biochemical processes in rice, including alterations in phytohormone profiles and metabolite dynamics, ultimately leading to substantial yield losses [5]. Understanding the mechanisms underlying heat-induced yield decline is thus imperative to safeguarding food systems in a warming climate. Heat stress impacts rice at multiple developmental stages, but the reproductive phase is particularly vulnerable [6,7]. Elevated temperatures during flowering and grain filling impair pollen viability, spikelet fertility, and carbohydrate translocation, resulting in reduced grain number and quality [8,9,10].

Based on molecular weight, heat shock proteins (HSPs) can be classified into large HSPs, HSP90, HSP70, HSP60, HSP40, and small heat shock proteins (sHSPs) [11,12]. HSPs are closely associated with plant thermotolerance, as knockout or overexpression of HSP genes has been shown to significantly alter heat tolerance in plants [13]. Hsp101, a member of the HSP100 family, acts as a key regulator of basal thermotolerance in rice by stabilizing the abundance of HSA32, a 32 kDa heat-stress-associated protein, in seeds and heat-stressed seedlings. This functional interplay between Hsp101 and HSA32 is critical for maintaining proteostasis under thermal stress, as evidenced by the marked reduction in Hsp101 levels and severely compromised basal thermotolerance observed in HSA32-knockout seeds compared to wild-type controls [14].

The *Hsp101-1* allele, identified as a novel genetic resource derived from neo-tetraploid rice, significantly enhanced the seed-setting rate in neo-tetraploid rice, highlighting its potential for rice breeding programs [15]. To elucidate *Hsp101-1*-mediated thermotolerance mechanisms, we generated transgenic rice lines overexpressing *Hsp101-1* and hypothesized that *Hsp101-1* orchestrates downstream transcriptional networks to stabilize reproductive development under heat stress. During the reproductive stage, flag leaves and panicles (focusing on spikelets) were sampled at six time points post heat stress (40 °C) for transcriptome sequencing. WGCNA was employed to (1) identify *Hsp101-1*-associated heat-responsive modules and hub genes; (2) map regulatory pathways linking *Hsp101-1* to thermotolerance; and (3) decipher spatiotemporal coordination of tissue resilience mechanisms.

This study establishes *Hsp101-1* as a central regulator that preemptively stabilizes stress-responsive transcriptomes. We demonstrate its role as a hub gene coordinating transcriptional reprogramming, thereby reducing the activation in heat-response pathways. The results revealed *Hsp101-1* regulatory networks during reproductive heat stress, offering strategic targets for climate-resilient rice breeding.

## 2. Materials and Methods

### 2.1. Cultivation, Heat Shock Treatment, and Transcriptome Sampling of Rice Materials

The OE transgenic lines were generated by cloning the full-length coding sequence of *Hsp101-1* into the expression vector pBWA(V)HS. Genetic transformation was conducted at Wuhan Boyuan Biotechnology Co., Ltd. (Wuhan, China). The vector was transformed into the *indica* rice landrace Huangyezhan via Agrobacterium-mediated transformation. Three independent T0 transgenic lines were selected, and the T2 generation was used for the heat stress treatments. WT and OE transgenic rice lines were cultivated in the experimental field of the Guangdong Academy of Agricultural Sciences. After the tillering stage, plants were transferred to walk-in growth chambers with controlled light and temperature conditions. Growth conditions were maintained at 28 °C with 75% relative humidity. Light intensity was set at 25,000 lx during the daytime (06:00–17:00) and 0 lx at night (18:00–05:00). Heat treatment of 42 °C started from 9:00 after 5 days of cultivation. Heat shock treatment was initiated during the meiosis stage, with a temperature set at 40 °C. Samples were collected from flag leaves and spikelets at the following time points: pre treatment (0 h, control, collected at 8:50) and 1 h (collected at 10:00), 2 h (collected at 11:00), 4 h (collected at 13:00), 8 h (collected at 17:00), and 24 h (collected at 9:00 next day) post treatment [16]. Immediately after collection, tissues were frozen in liquid nitrogen and stored at −80 °C in a refrigerator for subsequent RNA sequencing and transcriptomic analysis. This sampling strategy ensured the capture of dynamic transcriptional changes across critical phases of the heat stress response.

### 2.2. Transcriptome Sequencing

The transcriptome sequencing procedure encompassed the following steps: (1) RNA extraction and sample quality control: The RNA of all samples was extracted by using the Tiangen DP411 kit/TRIzol kit, which was purchased from Tiangen Biotech (Beijing) Co., Ltd., Beijing, China. RNA integrity and purity were verified by Nanodrop2000 and Agient2100 for concentration and integrity. (2) mRNA enrichment: Eukaryotic mRNA was isolated using Oligo(dT) magnetic beads. (3) Fragmentation: Purified mRNA was randomly fragmented using fragmentation buffer. (4) cDNA library preparation: Fragmented mRNA underwent end repair, 3′ adenylation (A-tailing), and adapter ligation. (5) Size selection: cDNA fragments of appropriate sizes were selected for PCR amplification according to the Illumina guidance (https://www.illumina.com/, accessed on 10 April 2023). (6) Library quantification: Library quality was inspected using the Qsep-400 method. (7) Effective concentration validation: qPCR was performed to accurately quantify the library’s effective concentration, ensuring sequencing accuracy. (8) Sequencing: Libraries were sequenced on an Illumina platform (Illumina NovaSeq 6000, https://www.illumina.com/, accessed on 10 April 2023) with paired-end 150 bp reads. This standardized workflow ensured high-quality transcriptomic data acquisition for subsequent differential expression and co-expression network analyses.

### 2.3. Analysis of RNA Sequencing Data

Raw sequencing data were first subjected to quality assessment and low-quality read filtering using fastp [17]. High-quality reads were then aligned to the MSU7 reference genome (*Oryza sativa japonica* cv. Nipponbare) [18] using the STAR software (v2.7.1a) [19]. Gene expression quantification was performed with RSEM (v1.3.1) [20], generating a Transcripts Per Kilobase Million (TPMs) expression matrix. Differential expression analysis was conducted using DESeq2 [21], with significant DEGs identified based on thresholds of |log2(fold change)| > 1 and adjusted *p*-value < 0.05. Heatmaps visualizing expression patterns were generated using TBtools v1.6 [22]. Gene Ontology (GO) enrichment analysis was performed using the ClusterProfiler package [23], highlighting biological processes and pathways significantly associated with heat stress responses. This pipeline ensured robust and reproducible transcriptomic profiling for downstream functional and co-expression network analyses.

### 2.4. WGCNA

The WGCNA pipeline [24] comprised the following steps: (1) Data Preprocessing: RNA-seq data were normalized using TPMs (Transcripts Per Million) and filtered to remove low-expression genes or those with excessive missing values, ensuring a robust expression matrix. (2) Network Construction: A weighted adjacency matrix was generated by applying a soft-thresholding power to the pairwise correlation matrix, preserving biologically meaningful connections. This matrix was further transformed into a Topological Overlap Matrix (TOM) to quantify network connectivity strength and reduce noise. (3) Module Identification: Genes were clustered into co-expression modules using the dynamic tree cut algorithm. Each module represented a functionally synergistic gene cluster (e.g., heat-stress-responsive modules). The expression profile of each module was summarized by its module eigengene (ME). (4) Module–Trait Association: Key modules were identified by calculating correlations between MEs and target traits (e.g., heat shock duration). For instance, the MEdarkslateblue module showed a significant positive correlation with transgenic thermotolerance. (5) Functional Annotation: KEGG pathway enrichment analysis [25] was performed on key modules to elucidate their biological roles (e.g., chaperone activity, ROS metabolism). Co-expression networks were visualized using Cytoscape v3.7.1 [26] to pinpoint hub genes acting as central regulatory nodes. This workflow systematically linked transcriptional coordination to phenotypic adaptation, providing insights into the modular architecture underlying rice heat stress resilience.

## 3. Results

### 3.1. DEGs Between WT and OE Lines After Heat Treatment

Transcriptome sequencing was conducted on flag leaves and spikelets of both WT and OE transgenic rice plants during the heading stage after a 40 °C heat shock treatment (Supplementary Table S1). Principal component analysis (PCA) was conducted using expression values, and the samples were clustered by tissues and heat treatment time (Supplementary Figure S1). Differential expression analysis revealed that in flag leaves, prior to heat treatment, there were 545 upregulated and 676 downregulated DEGs. Following heat shock, at 1, 2, 4, 8, and 24 h, the number of upregulated DEGs was 116, 117, 108, 199, and 189, respectively, while that of downregulated DEGs was 36, 226, 28, 90, and 88. In spikelets, the number of upregulated DEGs at the corresponding time points was 127, 102, 93, 110, and 167, and that of downregulated DEGs was 34, 94, 24, 10, and 21 (Table 1). The reduced number of DEGs post heat shock indicates that the overexpression of *Hsp101-1* already induced substantial transcriptional differences (OE vs. WT) prior to stress, suggesting that *Hsp101-1* may constitutively activate partial protective mechanisms. Furthermore, transgenic plants exhibited greater transcriptomic stability with reduced expression fluctuations under heat stress, implying that *Hsp101-1* could diminish the demand for stress-responsive activation in other genes by regulating core stress signaling pathways. This observation supports the hypothesis that *Hsp101-1* enhances thermotolerance through preemptive stabilization of gene expression and targeted modulation of critical stress-adaptive networks.

### 3.2. Functional Enrichment of DEGs

**Figure 1:**
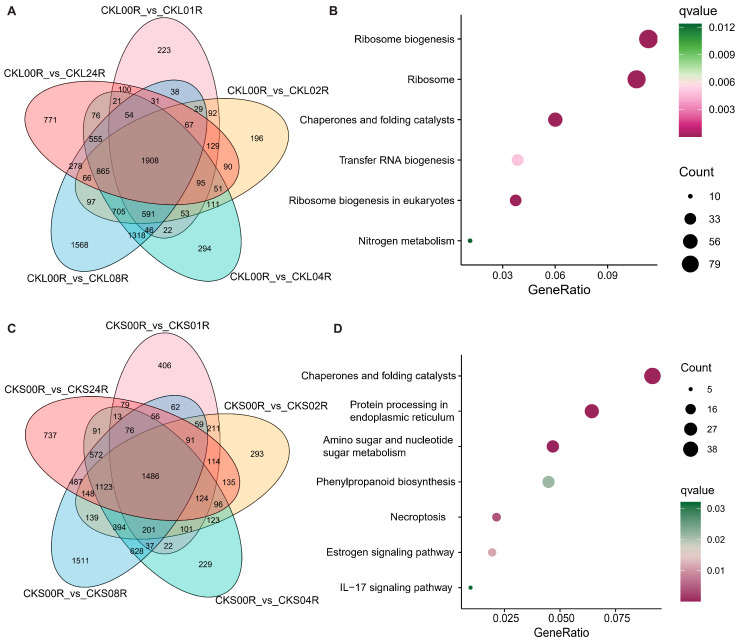
Differential expression analysis and Venn analysis of DEGs across distinct post-heat-shock time points versus pre treatment in wild-type line. Venn of DEGs in leaves (**A**) and spikelets (**C**) and their pathway enrichment results are illustrated in (**B**) and (**D**). The dot color and size in (**B**) and (**D**) represent the significance and gene counts in KEGG enrichment analysis.

**Figure 2:**
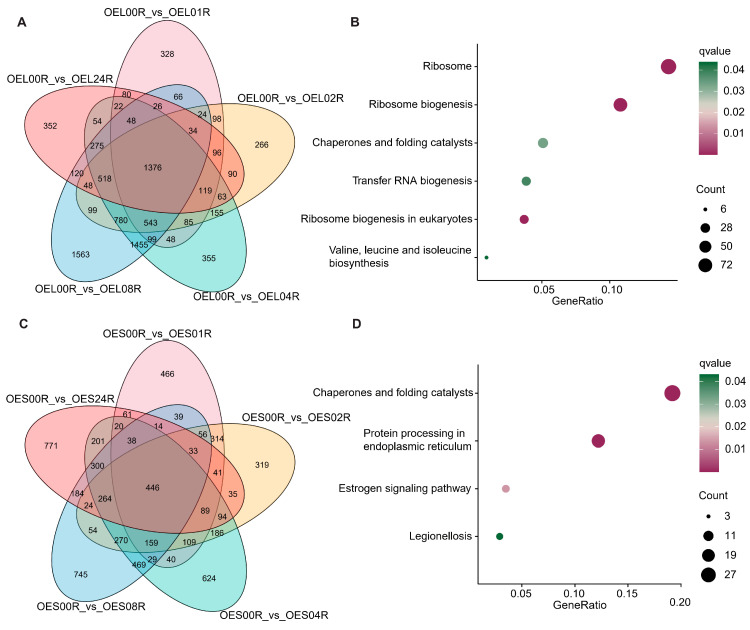
Differential expression analysis and Venn analysis of DEGs across distinct post-heat-shock time points versus pre treatment in *Hsp101-1* transgenic lines. Venn of DEGs in leaves (**A**) and spikelets (**C**) and their pathway enrichment results are illustrated in (**B**) and (**D**). The dot color and size in b and d represent the significance and gene counts in KEGG enrichment analysis.

Differential expression analysis and Venn analysis of DEGs across distinct post-heat-shock time points versus pre treatment were performed in both WT and transgenic lines. In WT flag leaves, 1906 common DEGs were identified across all five time points, with KEGG enrichment analysis revealing predominant involvement in pathways such as ribosome biogenesis, ribosomes, chaperones and folding catalysts, transfer RNA biogenesis, and ribosome biogenesis in eukaryotes. In WT spikelets, 1486 common DEGs were enriched in chaperones and folding catalysts, protein processing in the endoplasmic reticulum, amino sugar and nucleotide sugar metabolism, and phenylpropanoid biosynthesis (Figure 1). For transgenic flag leaves, 1376 common DEGs were similarly enriched in ribosome biogenesis, ribosomes, chaperones and folding catalysts, transfer RNA biogenesis, and eukaryotic ribosome biogenesis. Transgenic spikelets exhibited 1486 common DEGs, primarily associated with chaperones and folding catalysts, protein processing in the endoplasmic reticulum, and the estrogen signaling pathway (Figure 2). These results suggest that heat shock may directly disrupt ribosome structure or inhibit its function, prompting plants to upregulate ribosome-assembly-related genes to sustain protein synthesis capacity. As the most energy-demanding organelle, ribosome dynamics likely reflect optimized resource allocation under stress. Concurrently, chaperones may mitigate toxicity by assisting in the refolding or degradation of misfolded proteins. Collectively, these findings indicate that *Hsp101-1* enhances thermotolerance by alleviating activation pressure on core stress pathways, while the pronounced transcriptomic reprogramming in the WT line underscores the molecular basis of its heat sensitivity.

### 3.3. Identification of Co-Expression Modules by WGCNA

**Figure 3:**
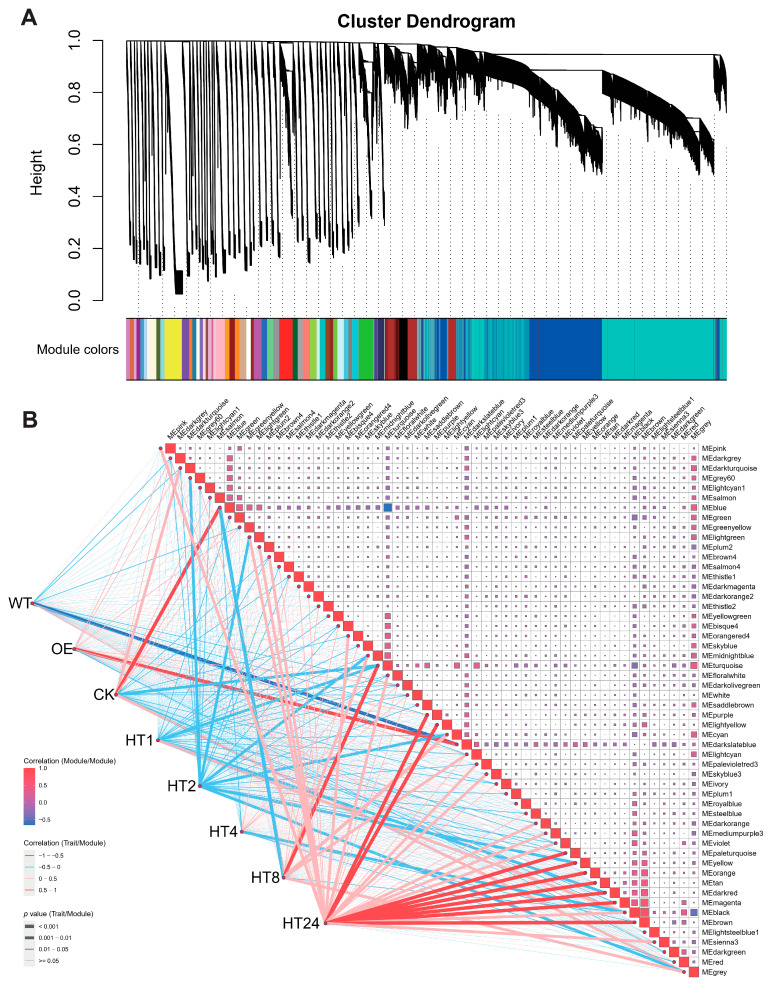
WGCNA of leaf transcriptome for WT and *Hsp101-1* transgenic lines. (**A**) Cluster dendrogram of WGCNA in flag leaf transcriptomes. (**B**) Correlation of gene modules for WT, OE, HT1 (1 h post heat treatment), HT2 (2 h post heat treatment), HT4 (4 h post heat treatment), HT8 (8 h post heat treatment), and HT24 (24 h post heat treatment); red and blue colors represent positive and negative correlations, and line width indicates *p*-values.

WGCNA of the flag leaf transcriptome grouped the genes into 54 modules (Figure 3, Supplementary Figure S2, Table S2). Among these, the MEdarkslateblue module was identified as the most relevant to the OE line, exhibiting a negative correlation with the WT line and a positive correlation with the OE line, suggesting that this module represents a key co-regulatory network (e.g., chaperones, protein-folding-related genes) critical for thermotolerance in transgenic lines. The negative correlation with the WT line implies impaired activation of these genes in the WT line under heat shock. Additionally, the MEblue module was positively correlated with pre-heat-shock conditions (CK), while the MEturquoise module showed a negative correlation with 1 h post treatment and a positive correlation with 8 h post treatment. The MEturquoise module was suppressed (negative correlation) at 1 h but activated (positive correlation) by 8 h, reflecting rapid transcriptional suppression during early heat stress and subsequent activation of repair mechanisms. By 24 h post treatment, the number of activated gene modules peaked, with at least nine modules showing significant positive activation. These dynamics highlight a phased transcriptional response to heat stress, combining immediate repression and delayed recovery processes, further supporting the role of *Hsp101-1* in stabilizing stress adaptation through coordinated regulation of critical gene networks.

**Figure 4:**
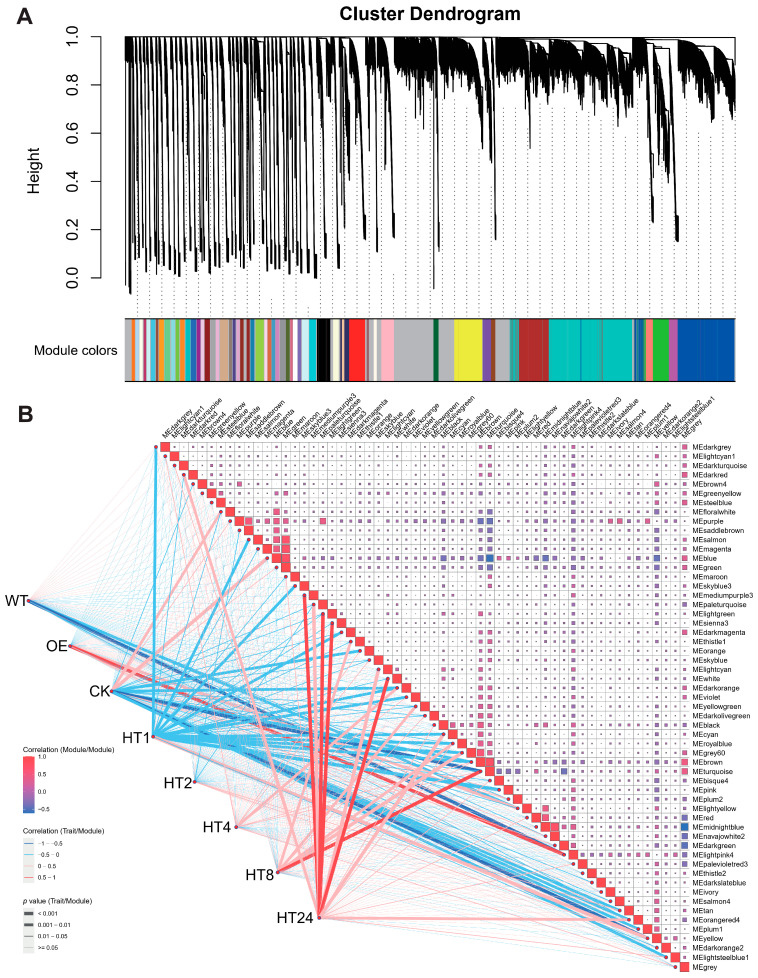
WGCNA of spikelet transcriptome for WT and *Hsp101-1* transgenic lines. (**A**) Cluster dendrogram of WGCNA in spikelet transcriptomes. (**B**) Correlation of gene modules for WT, OE, HT1 (1 h post heat treatment), HT2 (2 h post heat treatment), HT4 (4 h post heat treatment), HT8 (8 h post heat treatment). and HT24 (24 h post heat treatment); red and blue colors represent positive and negative correlations, and line width indicates *p*-values.

WGCNA of the spikelet transcriptome partitioned the genes into 57 modules (Figure 4, Supplementary Figure S3, Table S3). Among these, the MElightpink4 module was identified as significantly associated with both the WT and OE lines. Similar to the findings in flag leaves, this module likely contains genes critical to the coordinated regulatory network conferring thermotolerance in transgenic plants, as evidenced by its negative correlation with the WT line, implying that these genes fail to activate effectively in the WT line due to heat-induced damage. Additionally, the MEturquoise module exhibited a negative correlation with pre-heat-shock conditions (CK), indicating transcriptional repression under baseline conditions, but switched to a positive correlation (i.e., activation) at 8 h post heat treatment (HT8), mirroring the dynamic transition from early suppression to late-stage recovery observed in flag leaves. Consistent with the temporal pattern in flag leaves, the number of activated gene modules peaked at 24 h post treatment, with at least five modules showing robust positive activation. These results reinforce the hypothesis that *Hsp101-1* overexpression stabilizes stress-responsive transcriptional networks, enabling phased activation of repair mechanisms while mitigating the heat-induced dysregulation observed in the WT line.

### 3.4. Gene Network of the Key Gene Modules Between WT and OE Lines

**Figure 5:**
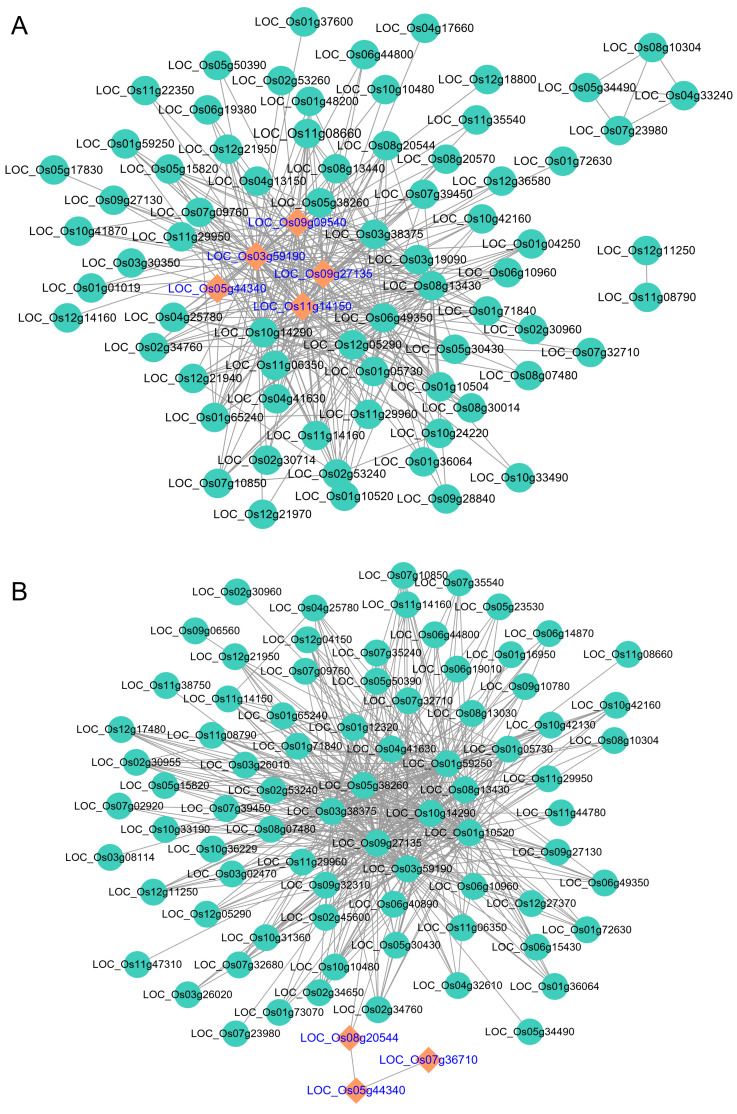
Gene network of the key gene modules between WT and OE lines. (**A**) *Hsp101-1* (*LOC_Os05g44340*) participates in gene module in flag leaves; (**B**) *Hsp101-1* (*LOC_Os05g44340*) participates in gene module in spikelets. The gene network was generated by WGCNA and illustrated in Cytoscape v3.7.1 software, and the genes directed linked to *Hsp101-1* are shown with orange rhombus nodes.

Further analysis of the key modules exhibiting negative correlations with the WT line but positive correlations with the OE line in both tissues revealed that the gene networks within these modules contained the *Hsp101-1* gene (LOC_Os05g44340) in both flag leaves and spikelets. Notably, four and two genes directly interacting with *Hsp101-1* were identified in flag leaves and spikelets, respectively (Figure 5). These findings strongly suggest that *Hsp101-1* acts as the central hub of these modules, orchestrating coordinated transcriptional responses critical to thermotolerance. The conserved presence of *Hsp101-1* as a core regulatory node across tissues underscores its pivotal role in stabilizing stress-adaptive networks in transgenic plants, while its absence or impaired connectivity in the WT line likely contributes to its heat-sensitive phenotype.

**Figure 6:**
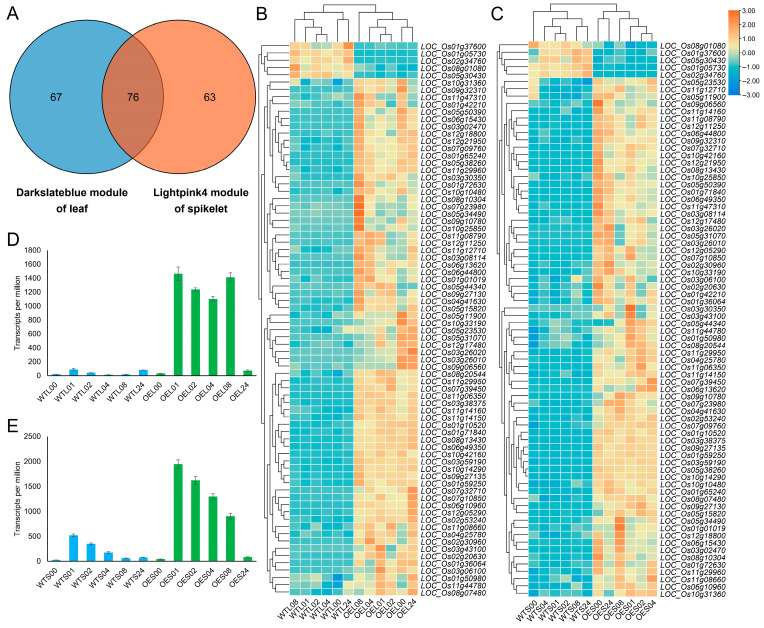
Venn and expression pattern analysis of the key modules in leaves and spikelets. (**A**) Venn of genes in Darkslateblue module of leaves and Lightpink4 module of spikelets. The numbers in plot indicates the count of common genes and each module. (**B**) Expression pattern of common genes in flag leaves. (**C**) Expression pattern of common genes in spikelets. Expression of *Hsp101-1* in flag leaves (**D**) and spikelets (**E**).

Venn analysis of genes from the two key modules identified 76 shared genes. Expression pattern analysis revealed that the majority (71/76) of these genes were upregulated in the OE line, suggesting that *Hsp101-1* may activate common downstream targets (e.g., molecular chaperones, ROS-scavenging enzymes) to establish a cross-tissue protective module, which cannot be effectively initiated in the WT line due to the absence of *Hsp101-1*. Expression profiling of *Hsp101-1* itself demonstrated low basal expression in the WT line but significant upregulation at 1, 2, 4, and 8 h post heat treatment, followed by a return to low levels by 24 h (Figure 6). This temporal pattern indicates that *Hsp101-1* rapidly responds to proteotoxic stress in early heat exposure, while its pre-activated chaperone network subsequently maintains cellular homeostasis in later stages, thereby preventing excessive energy expenditure. Collectively, these results highlight *Hsp101-1*’s dual-phase regulatory role: acute transcriptional induction to mitigate immediate damage, followed by systemic stabilization via preemptive stress-adaptive mechanisms.

## 4. Discussion

### 4.1. Core Mechanisms of Hsp101-1 in Plant Thermotolerance

This study demonstrates that overexpression of *Hsp101-1* significantly reduces the number of DEGs post heat shock, with downregulated genes decreasing by 83% in flag leaves and 77% in spikelets, indicating its role in alleviating the transcriptional stress burden through pre-activation of protective pathways. This aligns with the molecular chaperone function of Hsp101 as a member of the HSP100 family [27]. Persistent activation of ribosome biogenesis and chaperone pathways (e.g., 1906 common DEGs enriched in ribosome-related pathways in flag leaves) in the WT line reflects direct heat-induced disruption of protein synthesis systems. In contrast, transgenic lines likely stabilize ribosome structures via early *Hsp101-1* expression (peaking at 1–8 h post heat shock), thereby reducing the reliance on ribosome reconstruction. Notably, our findings that *Hsp101-1* preemptively mitigates transcriptional stress and stabilizes ribosomal machinery are consistent with studies in Arabidopsis, where HSP101 was shown to be crucial for resolving heat-induced translational arrest and promoting cellular recovery [13]. This mechanism parallels the role of Hsp101 in Arabidopsis, where *HSP101* affects the release of ribosomal protein mRNAs after heat treatment [28]. The findings may suggest that Hsp101-1 confers thermotolerance by preemptive mitigation of proteotoxic stress and targeted stabilization of critical cellular machinery. Notably, while the interaction between *Hsp101-1* and *HSA32* has been implicated in seedling thermotolerance [14], this study extends its regulatory role to the reproductive stage. The transient expression pattern of *Hsp101-1* in transgenic lines likely enables temporally specific activation of chaperone networks, facilitating rapid clearance of misfolded proteins during early heat stress while avoiding energy depletion caused by sustained overexpression. This dynamic regulatory mode explains its dual advantage in balancing high yield and thermotolerance.

### 4.2. The Molecular Basis of Transcriptome Stability

The pronounced transcriptional reprogramming in WT plants following heat shock (545 upregulated/676 downregulated DEGs in flag leaves and 127 upregulated/34 downregulated DEGs in spikelets) suggests a state of sustained stress, whereas transgenic lines exhibited significantly diminished gene expression fluctuations. The diminished transcriptional burden in transgenic lines reflects efficient preemptive adaptation through *Hsp101-1*-mediated network stabilization, thereby optimizing energy allocation for sustained growth under heat stress. WGCNA revealed that the MEdarkslateblue and MElightpink4 modules were positively correlated with *Hsp101-1* and enriched with chaperone proteins and ribosome biogenesis. These modules displayed negative correlations in the WT line, indicating that insufficient endogenous *Hsp101* expression prevents network activation. This phenomenon aligns with studies on the genetic basis of natural thermotolerance variation in rice, where impaired connectivity of key regulatory modules is a major contributor to heat sensitivity [29,30]. The failure of the WT line to engage these protective networks underscores *Hsp101-1*’s role as a molecular linchpin, bridging stress perception to adaptive transcriptional responses while mitigating the energetic costs of chronic stress adaptation observed in non-transgenic plants. The identification of 76 cross-tissue core genes (71 upregulated in OE line) suggests that these genes collaboratively enhance thermotolerance. The establishment of this multi-tiered network likely enables transgenic plants to restore homeostasis more rapidly post heat shock, thereby reducing the need to activate downstream repair pathways. This notion of efficient network stabilization is further supported by research in rice, which indicates that Snf2 family chromatin remodeling factors, responsive to abiotic stresses, can modulate gene expression and potentially contribute to transcriptome homeostasis under stress [31].

### 4.3. Potential Heat Memory in Modules After Hsp101-1 Expression Decline

The dynamic module patterns revealed by WGCNA (e.g., activation of the MEturquoise module at 8 h post heat shock) reflect stage-specific strategies in plant heat responses. Notably, even as *Hsp101-1* expression returned to baseline levels at 24 h, its downstream regulatory networks remained active (nine modules persistently activated). The alert state may be triggered by heat stress, and these modules that remain activated may be prepared for further heat damage. These heat shock memory benefits may confer critical fitness advantages under recurring heat stress. In Arabidopsis, *FORGETTER1* (*FGT1*) mediates heat stress memory by recruiting *SWI/SNF* chromatin remodeling complexes to maintain nucleosome-free regions at promoters of memory genes. This enables sustained H3K4me3 deposition by methyltransferases, poising genes for rapid reactivation upon recurrent stress [32]. Intriguingly, studies in *Arabidopsis* have revealed that HSP101 interacts with HSA32 at the post-transcriptional level to prolong heat acclimation memory, suggesting that protein complex persistence could be a mechanism underlying the sustained module activity we observed even after *Hsp101-1* transcript levels declined [33]. Thus, the alert state represents a resource-efficient adaptation to diurnal temperature fluctuations. However, whether plants employ analogous epigenetic or protein-based memory systems to maintain these activated networks requires further validation.

## 5. Conclusions

This study elucidates the molecular mechanisms by which *Hsp101-1* enhances thermotolerance in rice during the reproductive stage, offering critical insights for developing heat-resistant crops. By leveraging transcriptomic and co-expression network analyses, we demonstrate that *Hsp101-1* overexpression confers thermotolerance through two key strategies: (1) preemptive activation of protective pathways (e.g., chaperones, ribosome biogenesis) prior to heat stress, reducing the need for energy-intensive transcriptional overhauls during stress, and (2) tissue-specific coordination of stress responses, stabilizing ribosomal function in flag leaves to maintain photosynthesis while safeguarding spikelet development. Our findings highlight *Hsp101-1* as a central regulator of thermotolerance, orchestrating a conserved, cross-tissue defense network. This study not only advances the understanding of heat stress adaptation in rice but also provides an actionable framework for breeding climate-resilient varieties by targeting *Hsp101-1* and its synergistic gene modules. Such strategies could mitigate yield losses under global warming while balancing productivity and stress adaptation, ensuring food security in a warming world. Future studies should prioritize field validation of *Hsp101-1* lines under natural heat fluctuations and explore the epigenetic modifications underlying their sustained transcriptional memory, potentially unlocking novel breeding strategies for multi-stress resilience.

## Figures and Tables

**Table 1:** Count of DEGs between WT and OE after heat treatment.

Treatment	Up_Flag Leaf *^a^*	Down_Flag Leaf *^b^*	Up_Spikelet *^c^*	Down_Spikelet *^d^*
HT_00h *^e^*	545	676	910	1315
HT_01h *^e^*	116	36	127	34
HT_02h *^e^*	177	226	102	94
HT_04h *^e^*	108	28	93	24
HT_08h *^e^*	199	90	110	10
HT_24h *^e^*	189	88	167	21

The number of OE upregulated and downregulated DEGs in flag leaves and spikelets is presented in the table, with the corresponding column headers labeled as Up_Flag leaf ^(*a*)^, Down_Flag leaf ^(*b*)^, Up_Spikelet ^(*c*)^, and Down_Spikelet ^(*d*)^. ^(*e*)^ The labels HT_00h, HT_01h, HT_02h, HT_04h, HT_08h, and HT_24h denote heat treatment durations of 0, 1, 2, 4, 8, and 24 h, respectively.

## Data Availability

The raw reads of RNA resequencing are available in the NCBI Sequence Read Archive with accession ID PRJNA1290827. The sequences and annotations of reference genome MSU7 are available from the website http://rice.plantbiology.msu.edu/ (accessed on 10 August 2023).

## Supplementary Materials

The following supporting information can be downloaded at https://www.mdpi.com/article/10.3390/genes16091039/s1: Figure S1: Principal component analysis (PCA) of the 96 RNA sequencing samples. Figure S2: Value selection of power for WGCNA in flag leaves. Figure S3: Value selection of power for WGCNA in spikelets. Table S1: Sample information for RNA sequencing and mapping rate to the reference genome. Table S2: Gene modules classified by WGCNA in flag leaves. Table S3: Gene modules classified by WGCNA in spikelets.
