# Supplementary material for: Hsp101-1 Orchestrates Thermotolerance in Rice via Pre-Activated Transcriptional Networks and Modular Cross-Tissue Coordination
Source: Genes (Basel). 2025 Aug 31;16(9):1039. doi: 10.3390/genes16091039 (PMC12469619; doi:10.3390/genes16091039)
Supplement: Supplementary file 1 [file genes-16-01039-s001.zip › Supplementary Figures.pdf]

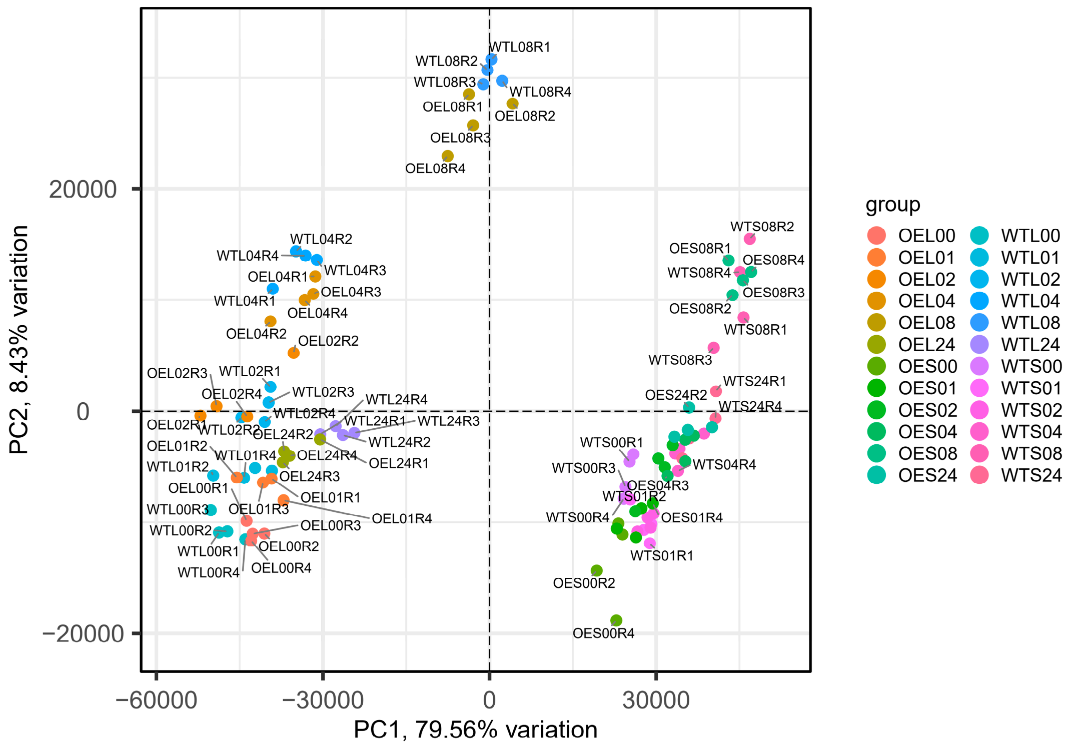

Figure S1. Principal component analysis (PCA) of the 96 RNA sequencing samples.

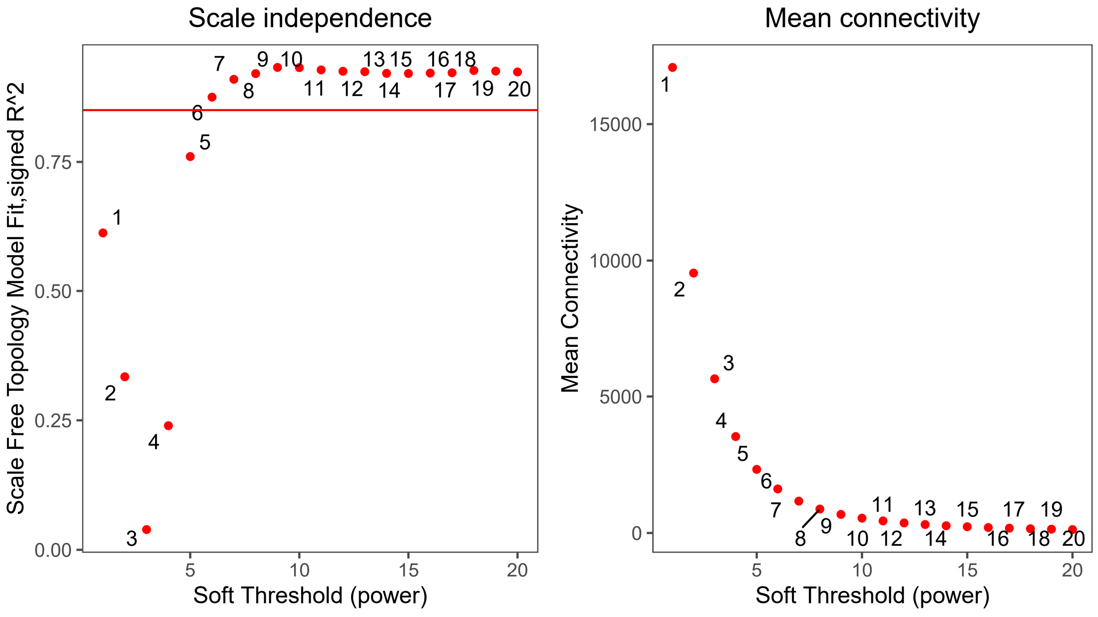

Figure S2. Value selection of power for WGCNA in flag leaf.

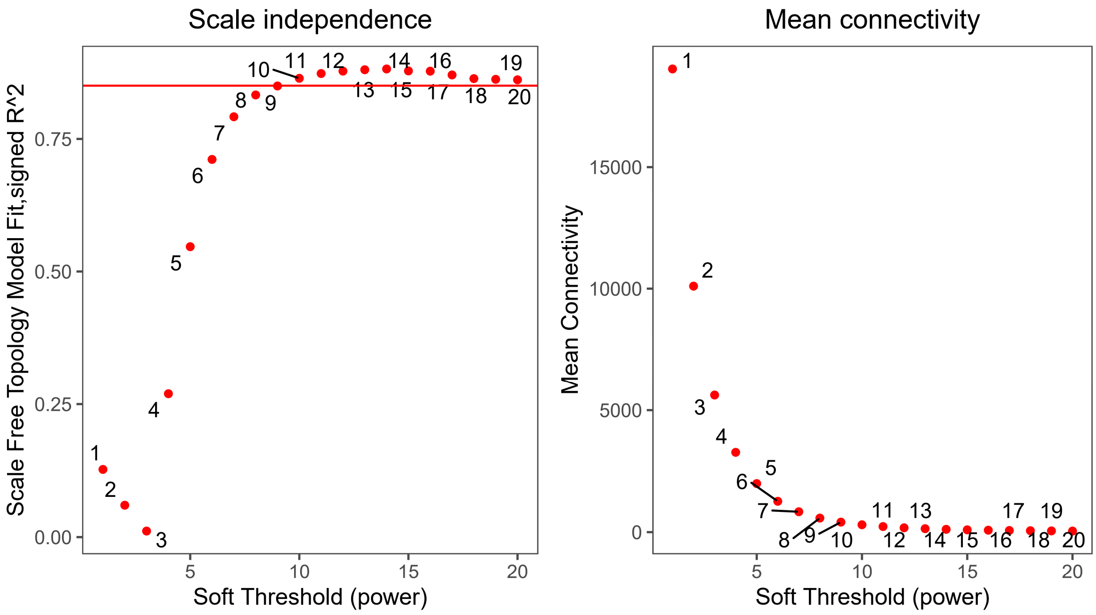

Figure S3. Value selection of power for WGCNA in spikelet.
